# Social and environmental risk factors for unintentional suffocation among infants in China: a descriptive analysis

**DOI:** 10.1186/s12887-021-02925-4

**Published:** 2021-10-22

**Authors:** Xue Yu, Lei Miao, Jun Zhu, Juan Liang, Li Dai, Xiaohong Li, Qi Li, Rui Rao, Chunhua Yuan, Yanping Wang, Chunhua He, Leni Kang

**Affiliations:** 1 National Office for Maternal and Child Health Surveillance of China, West China Second University Hospital, Sichuan University, Chengdu, Sichuan China; 2 Key Laboratory of Birth Defects and Related Diseases of Women and Children of the Ministry of Education, West China Second University Hospital, Sichuan University, Chengdu, China; 3 Department of Pediatrics, the people’s hospital of Leshan, Leshan, Sichuan China; 4 Department of Gynaecology and Obstetrics, Renshou Maternity and Child Health Care Hospital, Meishan, Sichuan China

**Keywords:** Unintentional suffocation, Risk factors, Infants, China

## Abstract

**Background:**

This retrospective study aimed to determine the epidemiological features of deaths caused by unintentional suffocation among infants in China.

**Methods:**

The data used in this study were obtained from China’s Under 5 Child Mortality Surveillance System (U5CMSS) from October 1, 2015, to September 30, 2016. A total of 377 children under 1 year of age who died from unintentional suffocation were included in the survey. Primary caregivers were interviewed individually using the Unintentional Suffocation Mortality among Children under 5 Questionnaire. EpiData was used to establish the database, and the results were analysed using SPSS 22.0.

**Results:**

Most (85.9%) unintentional infant suffocations occurred in rural areas, and 67.5% occurred in infants 0 to 3 months old. Among the primary caregivers of the infants, most (82.7%) had a junior middle school education or below, and 83.1% of them lacked unintentional suffocation first aid skills. Of the 377 unintentional suffocated-infant deaths, the causes of death were accidental suffocation and strangulation in bed (ASSB) (193, 51.2%), inhalation suffocation (154, 40.8%), other unintentional suffocation (6, 1.6%), and unknown (24, 6.4%). Among the infant deaths due to ASSB, overlaying (88.6%) was the most frequently reported circumstance. A total of 93.8% of cases reported occurred during co-sleeping/bed sharing with parents, and in 72.8% of the cases, the infants were covered with the same quilt as their parents. In our study, most inhalation suffocation deaths (88.3%) involved liquid food (such as breast milk and formula milk). A total of 80.5% of infant deaths reportedly occurred after eating; in 28.2% of those cases, the infants were held upright and patted by their caregivers, and 57.2% of them were laid down to sleep immediately after eating.

**Conclusions:**

To reduce the occurrence of unintentional suffocation, local government should strengthen knowledge and awareness of unintentional suffocation prevention and safety among parents and caregivers. Additionally, health care providers should educate parents and caregivers about safety issues of unintentional suffocation, and relevant policies should be introduced to provide environments and activities that reduce the risk of suffocation, such as promoting the Safe to Sleep Campaign. It is important to enhance the focus on infant unintentional suffocation as a health issue.

**Supplementary Information:**

The online version contains supplementary material available at 10.1186/s12887-021-02925-4.

## Background

Suffocation is a general term that includes many forms of asphyxia due to a scarcity of oxygen. It can happen when there is a lack of oxygen to breathe, or it can be due to an interruption of breathing due to obstructions of the external airways (smothering) or the internal airways (choking) [1]. Unintentional suffocation, including accidental suffocation and strangulation in bed and choking on food or other objects, causes serious injuries in children and is the leading cause of unintentional death in infants and toddlers [2]. Globally, there were more than 11,400 deaths in infants and toddlers aged 1–59 months reportedly due to unintentional suffocation in 2016 [3], causing great losses to families and society.

Accidental suffocation and strangulation in bed (ASSB) is a subset of sudden unexpected infant death (SUID); the mechanisms of ASSB are diverse and include the wedging of the child between objects, strangulation from cords or ties, suffocation by soft bedding, or overlaying by an adult or child [4–6]. A substantial proportion of these causes of death are related to the presence of objects in the sleep environment, suggesting that ASSB deaths can be prevented through the promotion of safe sleeping environments [7]. Inhalation suffocation, which is caused by choking on food or other objects, is the interruption of respiration by internal obstruction of the airway, usually by food or small toys, in young children [8]. The consequences of foreign body aspiration, such as asphyxia and cardiopulmonary arrest, are very serious and can lead to death or adverse neurological sequele [9].

In China, there were nearly 3200 deaths in infants and toddlers aged 1–59 months reportedly due to unintentional suffocation, accounting for 28% of unintentional suffocation deaths worldwide in 2016. The relative mortality among young children in China (5.33 per 100,000) is far higher than that in developed countries, such as the US (3.31 per 100,000), Canada (1.2 per 100,000), and Australia (0.8 per 100,000, 3]. Additionally, 83.8% of suffocation-related deaths among children younger than 5 years occur in infancy, and suffocation accounts for 83.0% of injury-related infant deaths [10]. Therefore, the prevention of unintentional suffocation deaths can have an important effect on reducing the overall infant mortality rate. Most unintentional suffocation-related deaths can be prevented by well-known strategies [2]. China released the ‘Healthy China 2030 Planning Outline’ in 2016 with the goal of establishing a comprehensive injury monitoring system and developing guidelines and standards for strengthening the prevention and intervention of injuries. Prevention should be based on clear epidemiological evidence of the behaviour and factors increasing the probability of suffocation [11]. However, previous resources have been limited by the lack of specific information, with the injury described only as the cause of death. In our study, given the absence of national-level data, we conducted one-to-one interviews with primary caregivers of infants who suffered from unintentional suffocation from various parts of the country (registered in the National Maternal and Child Health Surveillance System) to elucidate the characteristics and risk factors for unintentional suffocation deaths among infants. Our analysis focused on improving the epidemiological understanding of these deaths, thereby strengthening our ability to design more effective prevention strategies.

## Methods

### Study subjects

This study used data obtained from the U5CMSS (China’s Under 5 Child Mortality Surveillance System), a population-based surveillance system for monitoring the death of children younger than 5 years. It covered a total population of approximately 44–47 million individuals across 334 representative districts, of which 124 were urban and 210 were rural, in 31 provinces in mainland China. It follows stringent procedures for data collection, reporting, auditing, and quality control, helping to reduce the risk of under-reporting. Further details about the U5CMSS are described elsewhere [12]. Causes of death were classified according to the World Health Organization International Classification of Diseases, Tenth Revision (ICD-10).

The survey period was October 1, 2015 to September 30, 2016, and the parents of 377 children under 1 year of age in surveillance districts who died from unintentional suffocation were included in the survey. The unintentional suffocation in the study included W75–W76, W78–W80, and W83–W84. The research team participated in the research design, questionnaire design, and data collection and was responsible for descriptive analysis and reporting.

### Questionnaire

We collected information about unintentional suffocation deaths from the Unintentional Suffocation Mortality among Children under 5 Questionnaire, which was designed by the Chinese National Health Commission and United Nations International Children’s Emergency Fund (UNICEF) to gain information on children under five who died due to unintentional suffocation. The questionnaire contains two parts: basic information about children and their families (information on children, families, and the caregiving situation) and the basic conditions of the injuries (factors of unintentional suffocation, caregiving situation when the injury occurred, and rescue condition of the injury). All respondents provided oral informed consent for their anonymised responses to be analysed and published.

### Data collection and quality control

Local health workers from each district branch of China’s Under 5 Child Mortality Surveillance System (U5CMSS) were responsible for the organization, implementation, and conduct of the survey as well as for quality control of the data and reporting of the results. The investigators were trained to ensure that they fully understood the survey instructions and corresponding notes. During the interview, one trained investigator was paired with one respondent; the investigator read the questionnaire description and corresponding notes aloud to the respondent before completing the questionnaire with information dictated by the respondent. The same investigator checked the completeness and reliability of the data after the interview. Finally, the completed questionnaires were submitted stepwise via district-level, county, prefecture, city, and provincial-level maternal and child health care centers to the National Office of Maternal and Child Health Surveillance. To ensure the quality of the investigation, interviews with respondents were conducted within three months of the child’s death, and the maternal and child health care institutions at every level reviewed the data in a timely manner.

### Statistical analysis

The questionnaires were double entered with logic and consistency checks. The cases were analyzed and categorized according to sociodemographic characteristics associated with infants, basic characteristics of primary caregivers, and environmental risk factors. Proportions were calculated to describe the main results. EpiData (version 3.1, The EpiData Association, Odense, Denmark) was used to establish the database, and the results were analyzed using SPSS 22.0 (IBM, Armonk, NY, USA).

## Results

### Epidemiology of infants who died due to unintentional suffocation in China

**Fig. 1:**
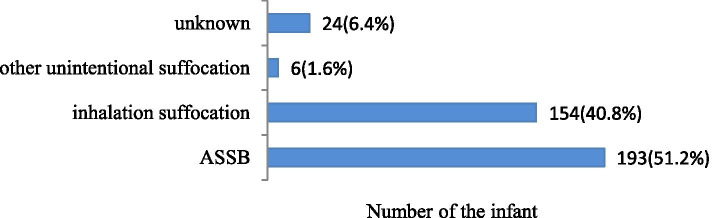
The mechanism of unintentional suffocation among the infant

Of the 377 unintentional suffocated-infant deaths, the causes of death were unintentional suffocation and strangulation in bed (ASSB) (193, 51.2%), inhalation suffocation (154, 40.8%), other unintentional suffocation (6, 1.6%), and unknown (24, 6.4%) (Fig. 1). Among these deaths, infants ≤3 months of age accounted for 67.5% of the cases; the 1-month-old group had the highest proportion of deaths, with 30.3% of deaths, and the 1–2-month-old group had the next highest proportion, with 24.8% of cases. The proportion of infant death decreased with age; only 13.2% of deaths occurred in the 6- to 12-month-old group. Among the unintentional suffocated-infant deaths, 56.8% of cases occurred in boys, 43.2% in girls, 13.3% in urban areas, and 86.2% in rural areas (Table 1).

**Table 1:** The descriptive characteristics of unintentional suffocations in the study

Characteristic	ASSB (n, %)	Inhalation suffocation (n, %)	Total (n, %)
the characteristic of unintentional suffocated infants			
Age (months)			
~ 1	46(23.8%)	59(38.2%)	105(30.3%)
~ 2	52(26.9%)	34(22.1%)	86(24.8%)
~ 3	29(15.0%)	14(9.1%)	43(12.4%)
~ 4	17(8.8%)	10(6.5%)	27(7.8%)
~ 5	14(7.3%)	10(6.5%)	24(6.9%)
~ 6	7(3.6%)	9(5.7%)	16(4.6%)
6 ~ 12	28(14.7%)	18(11.9%)	46(13.2%)
Sex			
Male	98(50.5%)	99(64.3%)	197(56.8%)
Female	95(49.5%)	55(35.7%)	150(43.2%)
Location			
urban	21(10.9%)	28(18.2%)	49(14.1%)
rural	172(89.1%)	126(81.8%)	298(85.9%)
the characteristic of infants’ primary caregivers			
Full-time care			
Yes	160(82.9%)	134(87.0%)	294(84.7%)
No	33(17.1%)	20(13.0%)	53(15.3%)
Relationship to child			
Only father	11(5.7%)	3(2.0%)	14(4.0%)
Only mother	71(36.8%)	59(38.3%)	130(37.5%)
Only parents	92(47.7%)	69(44.8%)	161(46.4%)
Father and grandparents	0(0.0%)	0(0.0%)	0(0.0%)
Mother and grandparents	3(1.6%)	6(3.9%)	9(2.6%)
Parents and grandparents	7(3.6%)	9(5.8%)	16(4.6%)
Only grandparents	7(3.6%)	7(4.5%)	14(4.0%)
Only Nanny	0(0.0%)	0(0.0%)	0(0.0%)
Not answered	2(1.0%)	1(0.7%)	3(0.9%)
Education level			
Unschooled	13(6.7%)	12(7.8%)	25(7.2%)
Primary schools	35(18.1%)	28(18.2%)	63(18.2%)
Middle schools	116(60.1%)	83(53.9%)	199(57.3%)
High school/technical school	11(5.8%)	14(9.1%)	25(7.2%)
Technical secondary school	6(3.1%)	2(1.3%)	8(2.3%)
Junior college or over	8(4.1%)	15(9.7%)	23(6.6%)
Not Answered	1(0.5%)	0(0.0%)	1(0.3%)
Unknown	3(1.6%)	0(0.0%)	3(0.9%)
Knowledge of first aid			
Yes	22(11.4%)	20(13.0%)	42(12.1%)
No	169(87.6%)	134(87.0%)	303(87.3%)
Not Answered	2(1.0%)	0(0.0)	2(0.6%)

### Basic information of primary caregivers of infants who died due to unintentional suffocation

#### Social features of primary caregivers

Most infants (84.7%) received full-time care (which is defined as the caregiver’s having no permanent job and only looking after one child) from their primary caregivers. The primary caregiver (which is defined as the caregiver who provided at least 6 h of caring per day) was only the mother in 37.5% of cases, and in 46.4% of cases, both parents were primary caregivers; grandparents took care of children in 4.0% of the cases. Among the primary caregivers, we found that most (82.7%) had a junior middle school education level or below, and only 2.3% had acquired some form of a bachelor’s degree (Table 1).

#### First aid knowledge of primary caregivers

The definition of unintentional suffocation first aid skills in this investigation was the ability of the primary caregivers to notice signs of suffocation and complete first aid for children, which included dislodging foreign objects using chest thrusts. Suffocation first aid skills were mostly lacking; the vast majority (87.3%) of primary caregivers reported having received no training (Table 1).

#### Activities of primary caregivers when unintentional suffocation occurs

At the time of unintentional suffocation, 81.0% of infants were with their primary caregivers. Regarding the primary caregivers who were with their infant at the time of suffocation, only 10.4% were supervising the infants. A smaller proportion (1.7%) was using their phone or socializing, and the majority (57.9%) was sleeping. It is worth noting that 70.0 and 42.2% of those primary caregivers were sleeping during infant deaths due to ASSB and inhalation suffocation, respectively, and 21.4% were taking care of the infants during inhalation suffocation (Table 2).

**Table 2:** The behavior of the primary caregiver at the time of unintentional suffocating

	ASSB (n, %)	Inhalation suffocation (n, %)	Total (n, %)
Who was with the child when the fatal accident occurred			
Primary caregivers	161(83.4%)	120(77.9%)	281(81.0%)
Other adults	9(4.7%)	5(3.3%)	14(4.0%)
Alone	13(6.7%)	19(12.3%)	32(9.2%)
Other children	2(1.0%)	1(0.7%)	3(0.9%)
Others	8(4.2%)	9(5.8%)	17(4.9%)
What was the primary caregiver doing when the injury occurred			
Doing housework	31(16.1%)	31(20.1%)	62(17.9%)
Watching TV	1(0.6%)	5(3.2%)	6(1.7%)
Sleeping	135(70.0%)	65(42.2%)	200(57.6%)
Eating	2(1.1%)	6(3.9%)	8(2.3%)
Looking after the infant	3(1.6%)	33(21.4%)	36(10.4%)
Others	6(3.2%)	2(1.3%)	8(2.3%)
Unknown	15(7.4%)	12(7.9%)	27(7.8%)

### Environmental features of infants who died due to different types of unintentional suffocation

**Fig. 2:**
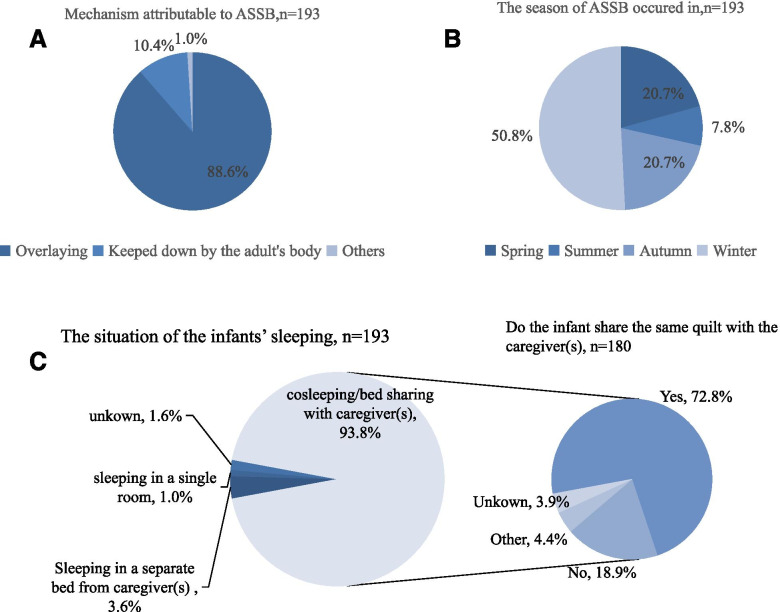
The characteristics of infants who died due to ASSB

Infants whose deaths were attributed to ASSB in our study had some noteworthy characteristics. Half (50.8%) of the ASSB cases occurred in winter, and only 7.8% occurred in summer. Overlaying was the most frequently reported circumstance, contributing to 88.6% of cases; 10.4% of the infants were overlayed by the adult’s body, and 1.0% of the cases were due to other causes. Of the 193 ASSB infant deaths, 93.8% reportedly occurred from co-sleeping/bed sharing with parents, and 72.8% of the infants who were co-sleeping/bed sharing with their parent were covered with the same quilt as their parent (Fig. 2).

**Fig. 3:**
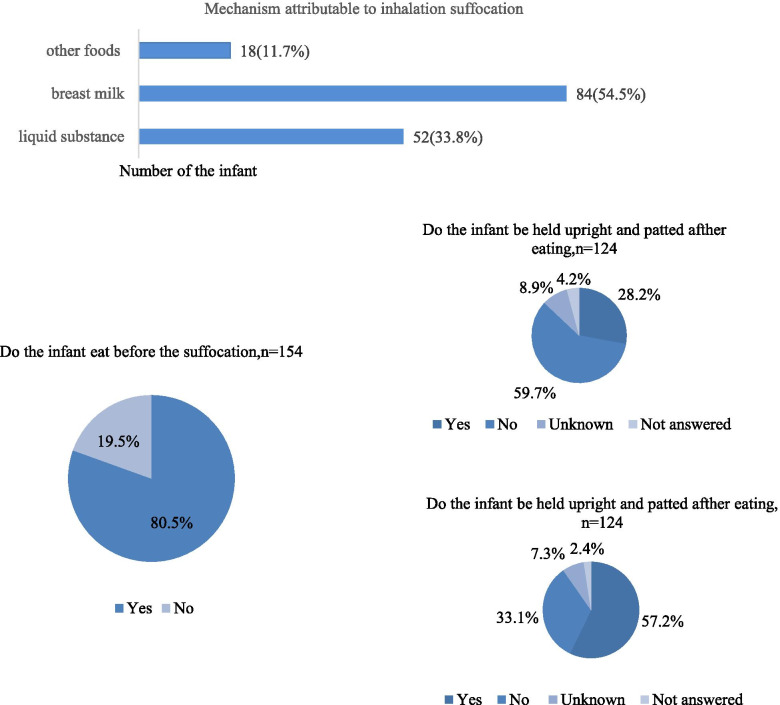
The characteristics of infants who died due to inhalation suffocation

We found that breast milk was the main cause of inhalation suffocation in infant deaths (54.5%), followed by a liquid substance (33.8%) and another semisolid or solid substance (11.7%). In our study, 80.5% of infant deaths due to inhalation suffocation reportedly occurred after eating; in 28.2% of these cases, the infants were held upright and patted by their caregivers, whereas 57.2% were put to sleep immediately after eating (Fig. 3).

## Discussion

In this study, we focused on unintentional suffocation among infants using data from the survey about the children’s characteristics, primary caregivers, and mechanism of unintentional suffocation deaths leading to the following findings. First, infants 0 to 3 months old (especially those 1 month old) who were living in rural areas had the highest mortality attributable to unintentional suffocation. Second, most primary caregivers (87.3%) had not received first aid training in suffocation, and most suffocated infants had caregivers who were less educated. Third, among infants who died due to ASSB, overlaying was the most frequently reported contributing factor, and most cases featured bed sharing, even sharing a quilt with the caregiver. Fourth, the most common substance of inhalation suffocation was breast milk and other liquid substances, and most infants who died due to inhalation suffocation did not receive protective measures, such as being held upright and patted by their caregivers and not being put to sleep immediately after eating. Therefore, our analysis suggests that several external factors influence the risk of infants’ dying due to unintentional suffocation: residence, age, unintentional suffocation safety training, and risky behaviors.

We found that the majority of infant deaths occurred in rural areas, consistent with previous studies [13, 14]. The difference in unintentional suffocation between rural and urban areas of China may be related to a lack of adequate care from caregivers due to farming duties or working outside, low safety awareness, and less knowledge among rural caretakers of children [15, 16]. One study showed that the childhood injury rate of left-behind children in rural areas (33.5%) was significantly higher than that of non-left-behind children (28.6%) [17]. In China, it is estimated that the number of left-behind children has skyrocketed to 61 million, which accounts for approximately 37% of all children in rural areas [18]. In addition, relatively weak prehospital aid and hospital treatment for children suffering from unintentional suffocation increased the risk that injuries that would result in mortality [19]. The results of the third National Health Service survey show that the density of health care organizations per 10 km2 is 1.41 vs. 0.21 (urban areas vs. rural areas), and the density of physicians and nurses per 1000 population is 3.8 and 3.8 vs. 1.0 and 0.7, respectively (urban areas vs. rural areas) [20]. Therefore, the government should pay more attention to improving the health of left-behind children, and more budget resources should be allocated to improving the construction of medical facilities and the medical treatment level.

The results showed that the highest percentage of cases occurred among infants who were 0 to 3 months old (especially 1 month old), and similar results have been reported in previous studies. For example, a study in the US showed that the unintentional suffocation deaths of infants aged 0–3 months accounted for nearly 60% of the cases [21]. In Australia, infants under 4 months were involved in more than 90% of unintentional suffocation deaths [22]. Regarding the high incidence of unintentional suffocation in infants, the most likely explanation is the imperfect physiological functions of newborns and infants and poor care by parents, which leads to asphyxia in bedding or improper feeding and asphyxia caused by foreign bodies in the respiratory tract [23, 24]. In China, many parents and adult caretakers prefer to sleep with their children in the same bed, especially when the children are infants, where quilts and pillows cover the baby’s mouth and nose or the parents’ arms and legs oppress the baby’s respiratory tract at night, resulting in unintentional suffocation. Local communities should strengthen publicity and education about safety awareness of unintentional suffocation [25].

Within the injury prevention community, it is accepted that up to 90% of childhood injuries are both predictable and preventable [24]. Professional knowledge of suffocation first aid can greatly improve the prognosis, especially for inhalation suffocation, which prevents oxygen from getting to the lungs and brain, leading to brain damage or even death within four minutes [26]. However, many parents in China do not know how to provide first aid on the spot, and they often panic when their children have accidents and do not give first aid treatment [27]. In our study, we found that the majority (87.3%) of primary caregivers of the infants did not have knowledge of first aid. In Saudi Arabia, 94% of parents had low-to-moderate first aid knowledge [28]. In Nottingham, 75% of parents knew the correct first aid for choking [29]. Changing community knowledge and/or behaviour through educational programs has a great effect on decreasing morbidity, mortality, and cost after unintentional injuries [28]. In the US, more than twofold shorter hospital stays and fewer full-thickness injuries were observed after community education [30]. In California, anoxic encephalopathy is less prevalent among children who receive immediate resuscitation following the event [31]. Therefore, it is important to educate caregivers about suffocation first aid. Paediatric health care providers should encourage parents and other caregivers to learn cardiopulmonary resuscitation (CPR) and choking first aid, and they should offer anticipatory, age-appropriate guidance to prevent unintentional injuries [2].

Accidental suffocation and strangulation in bed (ASSB) shares many of the same characteristics as sudden infant death syndrome (SIDS) [32], and there are no criteria to distinguish ASSB death from SIDS death, not even by autopsy alone or a full case investigation [33, 34]. In the US, a parsimonious explanation for the increase in deaths attributed to suffocation is misclassification bias arising from a change over time in the diagnosis of SIDS [35]. Taylor et al. showed that in Australia (25%), New Zealand (43%), the US (15%), Canada (5%), England and Wales (4%), and Germany (1%), unexpected infant deaths are attributed to accidental suffocation and strangulation in bed [36]. In our study, we could not completely rule out a small number of cases of diagnosis errors, but there was a stringent procedure for quality control in NMCHSS helping to reduce these errors.

Among the infant deaths due to accidental suffocation and strangulation, most occurred during sleeping, and risky infant behaviors were affected by unsafe sleeping environments. In our study, we found that the incidence of accidental suffocation and strangulation in bed was higher in winter, which is similar to American and Japanese reports [21, 37] We also found that 93.8% of cases reportedly experienced accidental suffocation and strangulation while the infants were cosleeping with their parents, which was similar to findings reported from previous surveys [38]. However, the extent of cosleeping in China is 57.8% [39], which is much higher than that in developed countries: 20–30% of mothers in the UK [40], 13.5–40% in the US [41], and 8.8–19% in New Zealand [9]. Takatsu et al. estimated that the mortality associated with infants who were cosleeping was 10.2 times higher than that of those who were not cosleeping [42]. During cosleeping, the infant’s mouth and nasal cavities can be obstructed by bedding or objects when the mother and infant are close to each other [42]. In our study, overlaying was the most frequently reported factor, contributing to 88.6% of cases. Therefore, creating a safe sleep environment for infants could substantially reduce infant suffocation deaths. The safest place for infants to sleep is on their backs on an unshared sleep surface, such as in a crib or bassinet, in the caregivers’ room, and without soft bedding (e.g., blankets, pillows, and other soft objects) in their sleep area [43]. Preventative efforts should target those at highest risk and focus on helping caregivers provide safer sleep environments.

In our study, inhalation suffocation was not a negligible phenomenon. It contributed to 40.3% of the deaths, and most of those deaths (88.3%) were due to liquid food, such as breast milk and formula milk. In addition to immature neuromuscular mechanisms of deglutition and airway protection, swallowing liquid food often contributes to choking during babyhood [44, 45]. There are many other risk factors. We found that 80.5% of inhalation suffocation cases occurred after eating, and half of the infants were put to sleep immediately after eating. A small proportion of caregivers held the infants upright and patted them. Therefore, proper feeding practices, including appropriate feeding time, correct feeding position, careful observation during the feeding process, and the expulsion of gastric gas after feeding, are critical for preventing infant inhalation suffocation. Governments and medical institutions should strengthen training on proper feeding for new mothers.

In view of the results of our study, it is important to enhance the focus on infant unintentional suffocation as a health issue and to integrate injury prevention efforts with a combination of education and policy. First, the government should strengthen publicity and education by disseminating unintentional suffocation prevention messages through channels, such as television, posters, and parent and caregiver learning experiences, to increase knowledge, attitudes, and behaviour change conducive to preventing injuries. Second, health care systems should play a critical role in educating parents and caregivers about safety issues of unintentional suffocation and encourage widespread CPR training among them. Last, the government should enforce policies to provide environments and activities that reduce the risk of suffocation, such as promoting the Safe to Sleep Campaign. Moreover, more attention should be given to injury prevention in rural areas.

## Conclusions

This cross-sectional study is the most extensive recent survey to examine the characteristics of unintentional suffocation deaths in infants in China. The risk factors for unintentional suffocation include a lack of comprehensive understanding of safety, poor awareness of prevention, and a lack of related policy. To reduce the occurrence of unintentional suffocation, local governments should strengthen knowledge and awareness of unintentional suffocation prevention and safety among parents and caregivers, health care providers should educate parents and caregivers about safety issues relating to unintentional suffocation, and relevant policies should be introduced to provide environments and activities that reduce the risk of suffocation, such as promoting the Safe to Sleep Campaign.

## Supplementary Information

**Additional file 1.**

## Data Availability

This study used data from the NMCHSS. This system was coestablished by the National Health and Family Planning Commission of the People Republic of China and Sichuan University, and it is owned by the National Health and Family Planning Commission of the People Republic of China. The researchers did not obtain consent to publicly share the data. The deidentified data set is available upon request to interested researchers. For data requests, please contact the Department of Science and Technology of West China Second University Hospital, Sichuan University, at: fu2yuankjb@163.com. This department is in charge of all programs in the hospital, including data management. One staff member from the department (named Xian He) monitors this email address.

## Abbreviations

U5CMSS: China’s under 5 Child Mortality Surveillance System
UNICEF: United Nations International Children’s Emergency Found
ICD-10: the World Health Organization International Classification of Dieases, Tenth Revision
ASSB: Accidental suffocation and strangulation in bed
SUID: Sudden unexpected infant deaths
