# Supplementary material for: Social and environmental risk factors for unintentional suffocation among infants in China: a descriptive analysis
Source: BMC Pediatr. 2021 Oct 22;21:465. doi: 10.1186/s12887-021-02925-4 (PMC8532275; doi:10.1186/s12887-021-02925-4)
Supplement: Supplementary file 1 — Additional file 1. [file 12887_2021_2925_MOESM1_ESM.docx]

Unintentional Suffocation Mortality among Children under 5 Questionnaire

Starting time（24-hour）: □□:□□

Ending time: □□:□□

After the survey is completed, please check whether all items are complete and answer the following questions:

1. Whether the respondents cooperate with the survey: □01=Yes □02=No

□98=Others

2. According to the investigator, the reliability score of the data^[[1]](#footnote-1)^：

Investigator name: Work Unit:

Phone number : Date:

Area county name:

The relationship between respondents and children:

□01=Father □02=Mother □03=Grandparents □04=Brother/Sister

□05=Nanny □06=Preschool teacher □98=Others

Are respondents primary caregivers of children **^[[2]](#footnote-2)^**？

□01=Yes □02=No

**Survey location:**

□01=in the children’ home □02= in the neighbor's home

□03= Hospitals/clinics □98=Others

**Part I basic information of children and families**

**1 basic information of children：**

- 1. children's number^[[3]](#footnote-3)^：
  2. name:
  3. the date of birth： year month day
  4. Sex: □01= Male □02= Female □99=Unkown
  5. Ethnic groups of Children^^[[4]](#footnote-4)^^：

□01= Han □02= Zhuang □03=Hui  □04= Uighurs □05= Mongolian

□06= Tibetan □98=Others □99=Unkown

- 1. Registered address of the child^^[[5]](#footnote-5)^^： Province City County (district) Town (street) Village (Neighbourhood committee)
  2. Whether the child's current address is the registered address^[[6]](#footnote-6)^?

□01=Yes □02=No，the current address：

- 1. the duration of residence of the child at the registered address : year month day
  2. Do children attend kindergarten?

□01=Yes □02=No □99=Unkown

1.10 Has the child been diagnosed with mental illness or other chronic diseases?

□01=Epilepsy □02= Amniotic fluid and meconium aspiration syndrome

□03= Congenital pyloric obstruction □04= Respiratory disease □05= Not sick □98=Others □99=Unkown

1.11 Is the child taking medication in the 24 hours prior to the injury?

□01= Yes，drug name （unkown: 000）□02=No □99=Unkown

1.12 Is the child medically examined?

□01= Yes，the age : year month day ( unkown: 000)

□02=Never（to 2.1） □99=Unkown （to 2.1）

1.13  the recent medical examination:（unkown: 000）

（1）height: cm

（2）weight： kg

（3）vision：□01=Normal □02=Myopia，the degreet of left eye the degree of left eye □03= Unchecked □99=Unkown

（4）hearing: □01=Normal □02=Unormal □03= Unchecked □99=Unkown

**2 basic information of family**

2.1 basic household information (please fill in form 1-1-1) :

2.2 the total family income: yuan（unkown: 000）

2.3 the residence:

□01=City □02=Suburb □03=County □04=Rural □98=Others □99=Unkown

2.4 homeownership:

□01=Owning □02=Renting（annual rent yuan）98=Others □99=Unkown

Table **1-1-1** Basic household information^1^

| No. | Age | Sex  01=male 02=female | Nation  （1） | Relationship with children（2） | Education level（3） | Professional（4） | Smoking  （5） | Drinking  （6） | Physical condition（7） | Mental condition（8） | Do care for children  （01=Yes  02=No） | Caring | |
| --- | --- | --- | --- | --- | --- | --- | --- | --- | --- | --- | --- | --- | --- |
|  |  |  |  |  |  |  |  |  |  |  |  | Nursing time（01=07:00~18:00 02=19:00~06:00 03=24h 04= not fixed） | Whether to be the primary caregiver for the child（01=Yes  02=No） |
| 1 |  |  |  |  |  |  |  |  |  |  |  |  |  |
| 2 |  |  |  |  |  |  |  |  |  |  |  |  |  |
| 3 |  |  |  |  |  |  |  |  |  |  |  |  |  |
| 4 |  |  |  |  |  |  |  |  |  |  |  |  |  |
| 5 |  |  |  |  |  |  |  |  |  |  |  |  |  |
| 6 |  |  |  |  |  |  |  |  |  |  |  |  |  |
| 7 |  |  |  |  |  |  |  |  |  |  |  |  |  |
| 8 |  |  |  |  |  |  |  |  |  |  |  |  |  |

 Instructions：

（1）Nationa: 01= Han 02= Zhuang 03=Hui  04= Uighurs 05= Mongolian 06= Tibetan 98=Others

（2）Relationship with children: 01=Father 02=Mother 03=Grandparents 04=Brother/Sister 05=Nanny 98=Others

（3）Education level：01=never school 02= Primary school 03= Junior high school 04= High/technical school 05= Technical secondary /Secondary school

06= college 07= University and above 99=unkown

（4）Professional：01= Administration of institutions 02= Military police 03= Senior and middle managers in large and medium-sized enterprises (non-owner status) 04= Private entrepreneur 05= Professional technical personnel 06= Handle affairs personnel 07= Individual industrial and commercial households 08= Business service personnel (including drivers) 09= Manufacturing and construction workers 10= Agricultural laborer 11= students 12= Unschooled child(Preschool children, out-of-school children) 13= Retired person 14= Unemployed personnel 98=Others 99=unkown

（5）Smoking：01= often（Smoking more than 1 cigarette per day for continuous or cumulative 6 months）, 02= Once in a while（Smoking more than four times a week, but less than one cigarette a day on average） 03=never 99=unkown

（6）Drinking: 01= often（weekly drinking more than one time）, 02= Once in a while（2 to 3 times one month，beer>1，liquor >2） 03=never 99=unkown

（7）Physical condition：01=health 02= Sick, do not need long-term medication 03= Sick, need long-term medication 98=Others 99=unkown

（8）Mental condition：01=health 02= Sick, do not need long-term medication 03= Sick, need long-term medication 98=Others 99=unkown

**Basic information of domestic households：**only persons living and living with children, but parents of children must be filled in.

**Care:** only those who had cared for children responded to the survey in the last 6 months.

2.5 Housing:^^[[7]](#footnote-7)^^：（unkown: 000）

（1）Floor area: square meters

（2）The number of people：

（3）the floor：

2.6 Type of medical institution nearest to current address? Distance: km

（unkown: 000）

□01=Army hospital □02=Provincial hospital □03=Prefecture-level hospital □04=District hospital □05=Township health centers/community health service □06= Village clinic/community health station □07= Private clinic □98=Others □99=Unkown

**3 Nursing Condition:**

- 1. Is it a full-time caregiver? □01=Yes □02=No

3.2 Does the child live with his or her parents?

（1）Father：□01=Yes □02=No，reasons^①^

（2）Mother：□01=Yes □02=No，reasons^①^

① a＝Work outside b= death c= Divorce or separation d= Others e= Unkown

3.3 In the most recent month, the time parents spend with their children per week is: Father ，Mother .

01=7h 02=7 ~20h 03=21~34h 04=35h and above 99= Unkown

3.4  Feeding of children before the age of 1 year ^[[8]](#footnote-8)^：

3.4.1 When breast-feeding or artificial feeding, does the caregiver pay attention to check the child's face and expression?

□01=Yes □02=No □03=Never □99=Unkown

3.4.2 Whether the caregiver has given the child hard candy/peanut/melon seeds/jelly, etc

□01=Yes □02=No □03=Never □99=Unkown

3.4.3 After the child has eaten, does the caregiver hold the child upright and pat his or her back to help him or her burp?

□01=Yes □02=No □03=Never □99=Unkown

3.5 Please answer the following questions:

（If the child is too young to answer the following questions, please check: □88= not applicable , skipping to 3.6）

3.5.1 Does the child watch TV or talk while eating?

□01=Yes □02=No □99=Unkown

3.5.2 Do children like to eat snacks or chew other items at ordinary times?

□01=Yes □02=No □99=Unkown

3.5.3 Do children like to snack or chew other things before bed?

□01=Yes □02=No □99=Unkown

3.6 Sleep of children before the age of 1 year^^[[9]](#footnote-9)^^：

3.6.1 Who the child sleeps with？

□01=share a bed with parents (or caregivers), width ， number of people sharing a bed  ：

□02=Share room with parents but sleep in separate bed，width （shipping to 3.6.3）

□03=  single room sleeping alone，width of child bed （shipping to 3.6.3）

□98＝ others □99=Unkown（shipping to 3.6.3）

3.6.2 If the child sleeps in the same bed with another person, does the child cover the same bed with another person?

□01=Yes □02=No □99=Unkown

3.6.3 Mattress Types of children's beds:

□01=latex □02=spring □03=palm □04=cotton □05= no mattress

□98＝ others □99=Unkown

3.6.4 When the child sleeps, what habitual action is there? (Optional)

□01= quilt head □02= plush toy □03=No habitual action

□98＝ others □99=Unkown

3.6.5 The main sleeping position of children is:

□01=prone □02=supine □03=side □98＝ others □99=Unkown

3.6.6 Whether caregivers check to see how the child is sleeping at night？

□01=always □02= sometimes  □03= never □99= Unkown

3.7  In the last 6 months, does the caregiver consider the size of toys and their accessories when purchasing children's toys?

□01=always □02= sometimes  □03= never □99= Unkown

3.8 Has the caregiver placed pillows, stuffed toys and other fluffy items on the child's bed in the last 6 months?

□01=always □02= sometimes  □03= never □99= Unkown

3.9 In the last 6 months, do children habitually put novelty (such as bright colors) objects in their mouths?

□01=always □02= sometimes  □03= never □99= Unkown

3.10 Does the caregiver have knowledge of emergency rescue for suffocation (on-site demonstration judgment)? □01=Yes □02=No（shipping to 3.12）

3.11 The source from which caregivers acquire the knowledge of suffocation first aid is (after multiple selection)：

□01=Hospital publicity □02= Government/administrative publicity

□03=community/family area publicity □04= telephone consultation

□05= relatives/friends/colleagues  □06=books/newspapers/magazines

□07= radio/TV □08= Internet

□98=Others □99= Unkown

3.12 What do the caregivers think can be done to reduce and avoid the occurrence of suffocation events? (Optional)

□01= maintain monitoring of children at all times □02=Use effective monitoring methods (eg. tying bells on children) □03= Remove safety hazards that easily cause suffocation □04=Educate parents about the prevention of accidental suffocation □05=To popularize the knowledge of first aid for accidental suffocation to parents

□98=Others □99= Unkown

3.13 Has the child received any education on the safety of accidental suffocation?（If the child is under 1 year old, please check: □88=not applicable，skipping to PartII）

□01=Yes □02=No（skipping to PartII）

□99= Unkown（skipping to PartII）

3.14 if you have received safety education, the sources are :(multiple choices are available)

□01=parents□02= grandparents □03=kindergarten □98=Others □99= Unkown

**Part II. The basic situation of the injury**

1. **Elements of injury：**

1.1 When the injury occurred?

Year: Month: Day: Time: （24-hour）（unkown: 000）

1.2 When the child died?

Year: Month: Day: Time: （24-hour）（unkown: 000）

1.3 Location of the injury：

□01=Home^①^ （skip to 1.5） □02=School（includes kindergarten）^②^

□03=Street/Road □04=Sports place

□05=Factory/ Construction site □06=Farm（excludes Home） □07= Commercial place □98=Other （Please note）

□99=Unknown

① a=Bedroom b=Living room c=Kitchen d=Restroom e=Other room f= Balcony g=Yard

h=Other（Please note）i= Unknown

② a=Classroom b=Playground c=Outside d= Other（Please note）e= Unknown

- 1. If the injury did not occur at home, the distance between the place where the injury occurred and the home is： maters（unkown: 000）
  2. Location of the child died：

□01=the place of injury occured □02=Health facilities (Hospitals/Clinics/Health centers)

□03= On the way to doctor

□77=Refuse to answer □98=Other（Please note）

□99=Unknown

- 1. Children's activities when the injury occurs：

□01=Eating/Dining □02=Playing □03=On the way to school/kindergarten

□04=Sleeping □98= Other（Please note）  □99= Unknown

- 1. Is the injury caused by irresistible factors (such as natural disasters)？

□01=Yes，please note □02=No □99= Unknown

- 1. The main reason caused asphyxia：

□01= liquids，please note □02= fish bone or other bone

□03= Melon seeds, peanuts, hard candy, etc

□04=Jelly □05=Breast milk □06=Torys □07= held down by an adult

□08= Covered by clothing/quilt □09= Buried in soil □10=Plastic bags

□98= Other（Please note） □99= Unknown

- 1. Did the child eat (including snacks) before the event of suffocation?？

□01=Yes □02= Yes（skip to 1.10） □99=Unknown（skipping to 1.10）

- - 1. If breastfeeding, what is the breastfeeding position of the mother? (If not，skipping to 1.9.3）

□01=Sitting □02= lie on the side □03= Supine □04= lie prostrate □98= Other □99= Unknown

- - 1. Whether the mother is awake while breastfeeding？

□01=Awake and clear □02=Tired □03=Sleeping/Napping

□77=Refuse to answer □99= Unknown

- - 1. After eating, does the caregiver hold the child upright and pat to help him or her to hiccup?

□01= Yes □02= No □99= Unknown

- - 1. Whether the child sleeps immediately after eating？

□01= Yes □02= No（skip to 1.10） □99= Unknown（skipping to 1.10）

- - 1. After eating, the sleeping position of the child is：

□01= lie on the back □02= lie on the side □03= Supine

□98= Other（Please note） □99= Unknown

- 1. Before suffocation, did children play or move in confined spaces (such as warehouses without windows, basements, cars)？

□01= Yes □02= No □99= Unknown

1.11 The main manifestations of children before death are: (multiple choices available)

□01=Edgy □02=Bucking □03= sweating

□04= Nasal flaring/trident sign □05= Complexion/Lip Bluish □06= be found dead

□07= No obvious abnormality □77=Refuse to answer

□98=Other （Please note） □99= Unknown

1. **Child’s supervise at the time of injury：**

2.1 Who was the child with when the injury occurred? (Multiple choice)

□01=Main caregivers（skip to 2.3） □02=Other adults^①^ □03=Alone

□04=Other children^②^ ，其中年龄最大的 岁 □99= Unknown

① 01=Father 02=Mother 03=Grandparents 04=Siblings 98= Other （Please note） 99= Unknown

② 01= Siblings 02=Neighbors 03=Classmates 98= Other （Please note） 99= Unknown

- 1. The time between when the primary caregiver learns that the child has been injured and when the child has been injured is：

□01=Within 10 mins □02=10~29 mins □03=30~59 mins □04=1~4 hours

□05=5~23 hours □06=Above 24 hours □99= Unknown

2.3 What was the primary caregiver doing when the injury occurred?(Optional)

□01=Doing housework □02=Socialiazing/Phone calling

□03=WatchingTV/PlayingComputer games □04=Sleeping/Resting

□05=Dining □06=Supervise the child □07= Supervise other children

□08=Working □98= Other （Please note） □99= Unknown

- 1. Did the primary caregiver consume alcohol or drugs within 6 hours prior to the injury?

□01=Drinking^①^ ，alcohol consumption ml □02=Medicine，named

□03=None alcohol or medicine □99=Unknown

① types：a=Liquor equal and over 42° b= Liquor less than 42° c=Beer

d= Huangjiu e=Rice wine f=wine g= Barley wine

1. **Rescue condition of the injury:**

3.1 The type of medical and health institution nearest to the place where the injury occurred?

- 1. Distance: km（unkown: 000）

□01= Army hospital □02= Provincial hospital □03= Prefecture-level hospital

□04= District hospital □05= Township health centers/community health service centers

□06= Village clinic/community health station □07= Private clinics

□98= Other （Please note） □99= Unknown

- 1. Whether the child receives first aid at the scene of injury？

□01=Yes □02=No（skip to 3.3 □77=Refuse to answer（skipping to 3.3） □99= Unknown（skipping to 3.3）

- - 1. Whether someone dialed emergency number (e.g. 120 or hospital phone number)？

□01= Yes □02 □77= Refuse to answer □99= Unknown

- - 1. Who gave first aid to the injured on the spot？

□01=Main caregivers □02= Passerby □03=Friend/other family members

□04=Teacher of kindergartens □05=Policeman □06=Fireman □07=Health worker

□77= Refuse to answer □98= Other （Please note） □99= Unknown

- 1. Whether the child went to the doctor after the injury occurred？

□01=Yes，at Year: Month: Day: Time: （24-hour）

□02=No

- 1. Treatment after the occurrence of the injury, and describe the order of treatment or treatment (fill in Table 1-1-2)：

Table 1-1-2 **The information of treatment**

| **Types** | **Health institutions types**（1） | | **Order^[[10]](#footnote-10)^** | **Distance**  （km）^[[11]](#footnote-11)^ | **Transports**  （2）^17^ | **Time costs**  （mins） | **Time of treatment**  （hours） | **Days of hospitalized**  （days） | **Treatment fees**  （Yuan） |
| --- | --- | --- | --- | --- | --- | --- | --- | --- | --- |
| □1.Untreated（skip to 3.8）^[[12]](#footnote-12)^ | | **—** | **—** | **—** | **—** | **—** | **—** | **—** | **—** |
| 2.Self-handling (on site) | | **—** |  | **—** | **—** | **—** |  | **—** |  |
| 3. Medical personnel handling（on site）^[[13]](#footnote-13)^ | | **—** |  |  |  |  |  | **—** |  |
| 4.Health institutions 1 | |  |  |  |  |  |  |  |  |
| 5. Health institutions 2 | |  |  |  |  |  |  |  |  |
| 6. Health institutions 3 | |  |  |  |  |  |  |  |  |

**Code description:**

（1）Health institutions types：01= Army hospital 02= Provincial hospital 03= Prefecture-level hospital

04= District hospital 05= Township health centers/community health service centers

06= Village clinic/community health station 07= Private clinics 98= Other （Please note）

99= Unknown

（2）Transports types：01=Walking 02=Biking 03=Motorcycling 04=Anbulance 05=Bus

06=Cars 07=Taxi 77=refuse to answer 98= Other （Please note） 99= Unknown

Total costs of treatment： Yuan，Reimbursement from parent company Yuan，Indemnification by the responsible party Yuan，Insurance company indemnity Yuan，Self-costs Yuan

Total transport costs（The total cost of transportation to and from a health facility due to an injury, including the cost of an ambulance and the total cost of transportation for the caregiver to care for the injured person）： Yuan

- 1. Total expenses for the nursing： Yuan
  2. The main reasons for not being treated or taken to hospital are：

□01=Died on site □02= Traffic inconvenience □03= Didn't have enough time

□04= Economic difficulties  □05= Local customs □77= Refuse to answer

□98= Other （Please note）  □99= unknown

1. **Reliability score**: According to the investigation conducted by the investigator, the full score is 100 points.80-100 is very reliable;60-79 are generally reliable;40-59 is not very reliable;40 is classified as unreliable. [↑](#footnote-ref-1)
2. **Primary caregiver：** a caregiver with no less than 6 hours of care per day. priority is given to investigating the primary caregivers who were present at the time of the accident;If the primary caregiver is not present at the time of the accident, priority is given to investigating the primary caregiver at the time of the accident (day or night).Where the above criteria are met, the child's mother will be given priority for investigation, which can be supplemented by other caregivers. [↑](#footnote-ref-2)
3. **children's number：**Please fill in the number on the child's death report card;Control Please fill in the number of matching cases. [↑](#footnote-ref-3)
4. **Nationality of children:** If the child has not yet entered the home, please fill in the nationality of the child's mother。 [↑](#footnote-ref-4)
5. **Household address:** If the child has not been registered yet, please fill in the household address of the child's mother. [↑](#footnote-ref-5)
6. **Current address:** If it is not a household address, please check "No" and fill in the current address on the horizontal line. The format is the same as the household address. [↑](#footnote-ref-6)
7. **Housing:** If you live in a multi-storey building, please fill in the total floor area and floor.For example, there are 3 floors, each floor is 100 square meters, the building area is 300 square meters, the floors are 1-3 floors. [↑](#footnote-ref-7)
8. **Feeding of children up to the age of 1 year:** fill in the general condition of children up to the age of 1 year. [↑](#footnote-ref-8)
9. **Sleep conditions of children before the age of 1 year:** please fill in the information of general conditions of children before the age of 1 year. [↑](#footnote-ref-9)
10. **Sequence:** Refers to the sequence of various processing types.For example, if you first dispose of it by yourself and then send it to the county hospital, you should fill in 1 in line 2, 2 in line 5 and leave the rest blank. [↑](#footnote-ref-10)
11. **Distance, transportation:** if there is a variety of processing type, the distance and transportation refers to a type to the next processing type of distance and the way of transportation, such as: treatment types to choose medical staff (live), medical institutions, medical institutions, 1, 2, then the distance and transportation in medical and health institutions 1 refers to the injury site and using the modes of transport, the distance to the agency 1 in the medical and health institutions 2 distance and transportation refers to the distance from the mechanism of 1 to 2 and transportation. [↑](#footnote-ref-11)
12. **Not being treated or taken to hospital：**After an injury occurs to a child, if nothing has been done, please tick the box above and jump to 3.8. [↑](#footnote-ref-12)
13. **Medical personnel handling（on site）：**The bank respectively filled in the distance of medical personnel to the scene, mode of transportation, times of transfer, journey time, treatment time and treatment cost. [↑](#footnote-ref-13)
